# Comparison of dynamic compression system versus multiple cancellous screws in the treatment of femoral neck fractures in young adults

**DOI:** 10.1186/s13018-024-04913-7

**Published:** 2024-07-22

**Authors:** Omar Aljasim, Can Yener, Nadir Özkayın

**Affiliations:** 1 Department of Orthopedic Surgery, Ege University Medical Faculty Hospital, İzmir, Turkey; 2 Department of Orthopedic Surgery, Hand, Microsurgery, Orthopaedics and Traumatology (EMOT) Hospital, İzmir, Turkey

**Keywords:** Femoral neck fracture, Internal fixation, Multiple cancellous screws, Dynamic compression system

## Abstract

**Introduction:**

Femoral neck fractures have posed a significant global healthcare challenge and had notable impacts on the quality of life. Current treatment strategies for femoral neck fractures in young individuals have varied, emphasizing the need for optimal fixation methods. This study compared the clinical and radiological outcomes of the dynamic compression system (DCS) and multiple cancellous screws (MCS) methods.

**Methods:**

This retrospective study included a total of 275 young adults with fresh femoral neck fractures treated with DCS and MCS. A matching analysis with a 1:1 ratio based on age, gender, fracture classification, and reduction quality was conducted. Demographic data were recorded, and comparisons were made according to follow-up time (FUT), hospitalization period, operation duration, femoral neck shortening, caput-collum-diaphysis (CCD) angle, Harris Hip Score (HHS), and post-operative complications.

**Results:**

A total of 42 fractures were matched with a median age of 42 years (range, 22–48). In the DCS group, vertical neck shortening (median 1.92) was significantly lower than that in the MCS group (median 4.53) (*P* < 0.05). In the DCS group, horizontal femoral neck shortening, resultant femoral neck shortening, the amount of change in CCD angle, and HHS were 0.57 mm (0.43, 4.74 mm), 1.82 mm (0.40, 3.53 mm), 0.13° (-0.78°, 1.80°), and 91 (85–93), respectively. They were all non-significant than 1.00 mm (0.56, 6.23 mm), 2.74 mm (1.59, 6.71 mm), -0.18° (-1.11°,1.85°), and 91 (75, 93) in the MCS group, respectively (*P* > 0.05). There was no statistical difference in FUT, hospitalization period, operation time, and post-operative complications at the latest follow-up (*P* > 0.05). There were no complications such as pulmonary embolism, deep vein thrombosis, and incision infection reported.

**Conclusion:**

DCS and MCS demonstrated effectiveness in treating femoral neck fractures in young adults. The DCS implant provides additional stability in the vertical axis. A prospective randomized controlled study with a large sample size was needed to validate these findings.

**Supplementary Information:**

The online version contains supplementary material available at 10.1186/s13018-024-04913-7.

## Introduction

Hip fractures have emerged as a growing global healthcare concern, causing a substantial decline in the overall quality of life [1]. Approximately half of all hip fractures were attributed to femoral neck fractures, with most incidents transpiring in old age individuals following low-energy trauma [2]. Femoral neck fractures were infrequent in young adults, typically arising from high-energy trauma [3]. Recent studies provided sufficient evidence to support the use of hip arthroplasty in elderly individuals with a displaced femoral neck fracture. These studies demonstrated a low complication and reoperation rate, with better functional outcomes compared to internal fixation [4]. On the other hand, preserving the natural hip anatomy and mechanics was considered a top priority in young patients with good bone quality [5].

Several implants had been developed over time for internal osteosynthesis of the femoral neck fractures. The conventional techniques for fixation still included the utilization of dynamic hip screw (DHS) and multiple cancellous screws (MCS) [6]. Targon femoral neck system (TFNS) was described as an alternative fixating device. Finite element analysis revealed that TFNS outperformed DHS and MCS in its ability to withstand shearing and rotational forces [7]. Nevertheless, no significant difference in the clinical outcomes and revision rates was observed [8]. The recently developed femoral neck system (FNS) integrated the benefits of both DHS and MCS. Clinical studies showed no significant differences in the fixation stability or the final functional outcomes [9]. In general, no consensus on the optimal treatment approach had been reached [6].

The Dynamic Compression System (DCS) was described as a novel fixation device that merged the characteristics of both MCS and DHS implants. DCS provided controlled dynamic compression by utilizing three screws to apply pressure parallel to the axial orientation of the femoral neck at the fracture site [10]. A study showed that DCS had superior functional and mechanical outcomes compared to MCS [11]. Other studies reported DCS as a less invasive device than DHS with a comparable complication rate [12]. Previous studies had a small sample size with non-identical baseline characteristics.

This retrospective case-control study aimed to assess the outcomes of two patient groups (DCS, MCS) with relatively identical baseline characteristics. It was hypothesized that the DCS would provide better mechanical stability than the MCS, minimizing postoperative femoral neck shortening and angular deformity. Additionally, it was presumed that increasing the stability would improve the functional outcomes.

## Materials and methods

### Study design and patients

This retrospective study was conducted at a single tertiary trauma center. Approval was obtained from the institutional ethics committee (19-12.1T/13), and written consent was obtained from all participants. Data were obtained from the electronic archive of Ege University Hospital using the ICD-10 code S72.0 for the fracture of the head and neck of the femur, according to the 10th revision of the International Statistical Classification of Diseases and Related Health Problems. A total of 688 patients with intracapsular hip fractures were admitted from January 2016 to November 2020. The inclusion criteria were as follows: (1) age range from 18 to 50 years, (2) patients could walk autonomously before the injury (3) fresh femoral neck fractures (less than 48 h), (4) patients treated with internal fixation, either DCS or inverted triangle MCS, (5) the follow-up time was at least 2 years, and (6) unilateral traumatic femoral neck fracture. The exclusion criteria were: (1) pathological fractures or fractures treated after 48 h, (2) patients with severe multiple traumas, or prior femur surgery, (3) musculoskeletal disorders, (4) patients treated with triangle MCS configuration, (5) fewer than 3 screws or more than 3 screws, and (6) convergence angle greater than 10°. Individuals with osteonecrosis, implant failure, or those requiring re-operation were included irrespective of whether their follow-up duration was less than 2 years. The patient population that met the inclusion criteria was subjected to a 1:1 matching based on five criteria: age, gender, reduction quality, Garden classification, and Pauwels classification. This study followed the STROBE guidelines for reporting observational studies. The study design, data collection, analysis, and reporting were conducted in accordance with these guidelines to ensure transparency, reproducibility, and accuracy of our findings [13].

### Surgical technique

All operations were conducted under either general or regional anaesthesia. Patients were positioned supine on an orthopaedic traction table. The fractured limb was placed in a slightly internally rotated and abducted position, with the affected hip elevated at an angle of 10°–15°. Intraoperative anteroposterior (AP) and lateral radiographs were obtained using a C-arm fluoroscopy machine. The quality of reduction was assessed using the Garden’s alignment index. On the AP radiograph, an angle of 160°-180° between the primary compressive trabeculae of the diaphysis and the neck was considered acceptable. The reduction was deemed acceptable on the lateral radiograph when the major trabeculae extended at a 180° angle to the femoral neck axis [14]. If the closed reduction manoeuvre failed to meet the Garden’s alignment index, an open reduction was performed through the Watson-Jones approach. Otherwise, a limited lateral approach was made approximately 5–10 cm below the greater trochanter. Subsequently, the tensor fasciae lata was incised, and the vastus lateralis muscle was retracted anteriorly, exposing the bone surface beneath the greater trochanter.

In the MCS group, a Kirschner guide wire was introduced under image intensifier control, parallel to the head-neck axis within 3 mm from the inferior endosteum of the femoral neck. Two superior wires were placed parallel to the first guide wire, forming an inverted triangular configuration. The posterior wire was placed within 3 mm from the endosteum of the posterior cortex. After determining the length of the screws, a cannulated drill with a diameter of 3.6 mm was used to drill over the wires. Three cannulated cancellous screws with a diameter of 7.0 mm were inserted over the wires and tightened. Washers were used in the proximal screws to prevent cortical penetration.

In the DCS group, an appropriate plate size was selected, and a dynamic compression plate was placed over the lateral cortex. Subsequently, Kirschner guide wires were introduced through the guide wire sleeve fixed in the plate. The positions of the guide wires were adjusted as mentioned previously. After measurement and drilling were conducted, three cannulated cancellous screws with a diameter of 7.0 mm were inserted and tightened. The locking set screws were not used in any patient.

In both groups, the screws were carefully and repeatedly tightened after the traction was released. Attention was directed toward ensuring that the threads of the screws were situated distal to the fracture line, and all screw tips were positioned approximately 5 mm below the subchondral bone.

### Postoperative management

The patients were permitted active-assisted mobilization once the pain was under control, with non-weight bearing on the affected limb. At the 3rd to 4th weeks post-surgery, patients were permitted to bear partial weight, and full weight-bearing clearance was allowed between the 8th and 10th weeks after the procedure. Patients underwent regular follow-ups at the 3rd week, 6th week, 3rd month, 6th month, the first year, and the second year after surgery. During each visit, AP and lateral radiographs were obtained.

### Measurement

Radiographs were independently assessed by two orthopaedic consultants. Fracture reduction quality was evaluated using a quantitative indicator based on displacement distance and the angle of deformity. Reduction was classified as excellent (less than 5° angulation in any plane or less than 2 mm displacement), good (5° to 10° angulation in any plane or 2 to 5 mm displacement), fair (10° to 20° angulation in any plane or 5 to 10 mm displacement) and poor (more than 20° angulation or more than 10 mm displacement) [15].

**Fig. 1:**
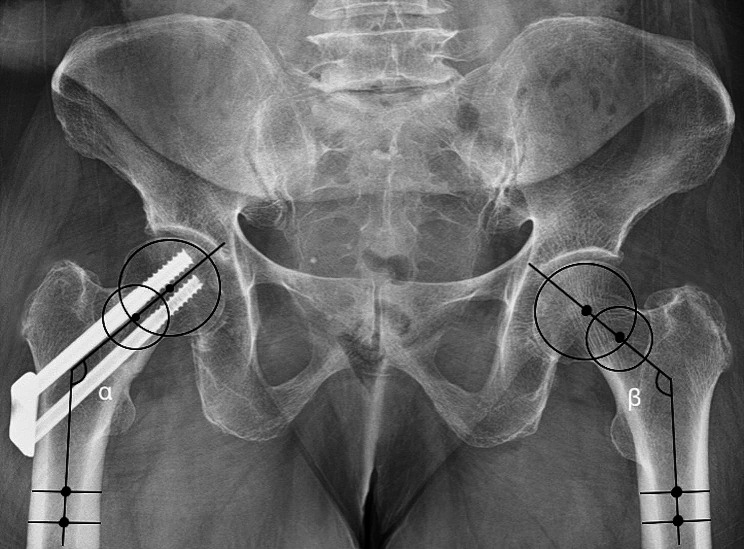
Measurement of caput-collum-diaphysis (CCD) angle. α: CCD angle of the fractured hip, β: CCD angle of the uninjured hip

The caput-collum-diaphysis (CCD) angle of the injured and non-injured sides was measured as described by Park et al. [16] on the AP radiographs (Fig. 1). Measurements were obtained from the immediate post-operative radiographs and from the final follow-up radiographs. CCD angle measurements were conducted using RadiAnt DICOM Viewer 2023.1 (Medixant Incorporated, Poznań, Poland). A negative number in the CCD readings indicates slightly valgus angulation compared with the unaffected side.

**Fig. 2:**
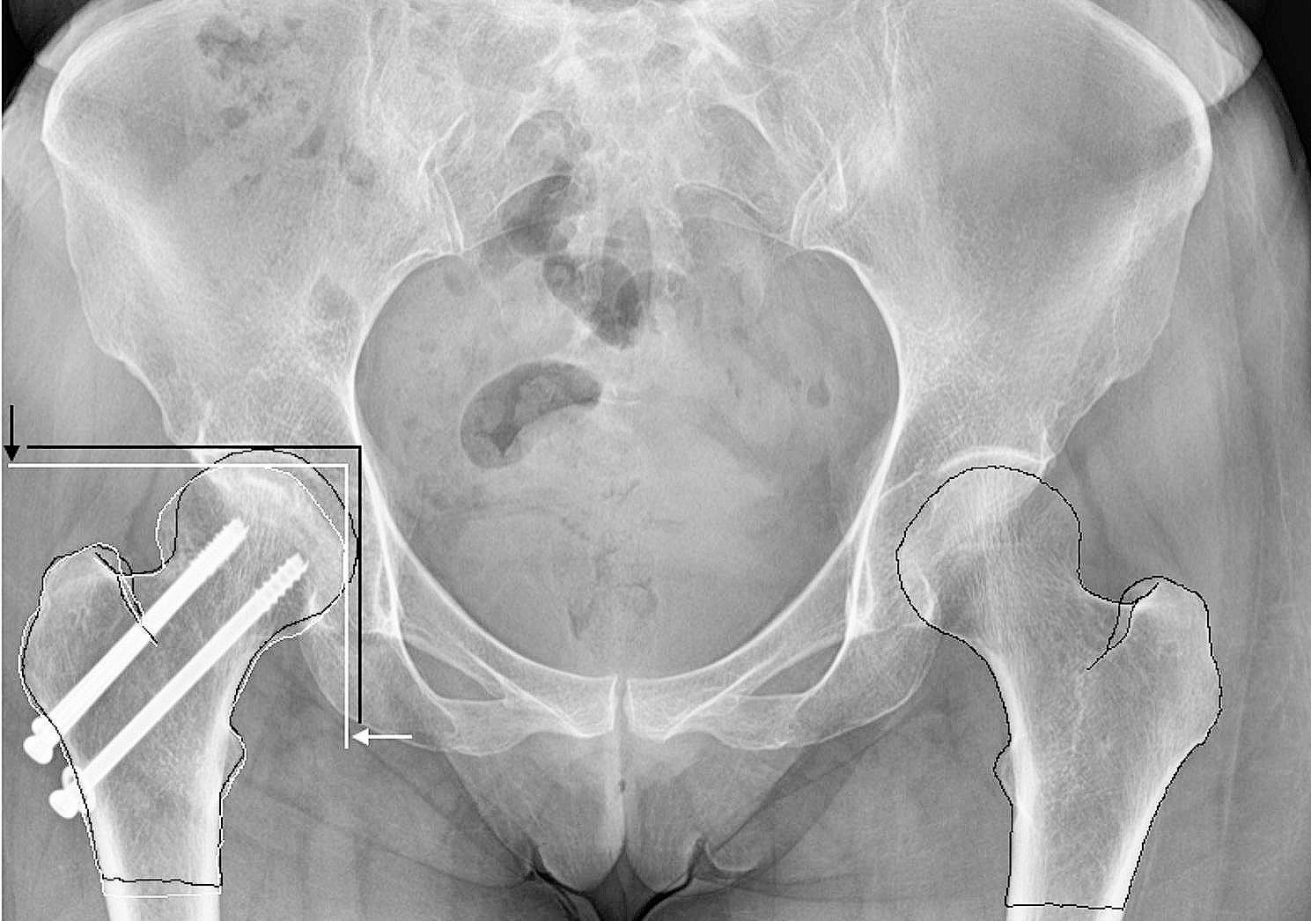
The template of the uninjured hip transposed over the operated side with outlining the operated hip. Vertical (Y axis) and horizontal (X axis) femoral neck shortening was measured as described by Zlowodski et al. [17]

Femoral neck shortening was measured on AP radiographs of the final follow-up visit. The uninjured contralateral proximal femur was measured using as a template to measure the femoral neck shortening of the fractured side as described by Zlowodzki et al. [17]. Femoral neck shortening was conducted in the vertical (Y axis) and horizontal plane (X axis) on digital AP radiographs. The amount of femoral neck shortening was calculated using Y axis, X axis and θ as the corresponding CCD angle as follows: Z = Y*sin(θ) + X*cos(θ), where Z is the femoral neck shortening vector [18]. Radiographic measurements were standardized using the known diameters of screws. Adobe Photoshop CC (Adobe Systems Incorporated, California, United States of America) was utilized for templating and distance measurements (Fig. 2).

The presence of posterior comminution was assessed by examining the pre-operative and early post-operative radiographies. Non-union was identified as the absence of radiographic healing progression at the 6th month [19]. Osteonecrosis of the femoral head was considered an outcome event if subchondral sclerosis or segmental collapse were observed [19].

The evaluation of hip function was performed by calculating the Harris Hip Score (HHS) at the latest follow-up visit [20, 21]. Operational time, duration of hospitalization, follow-up duration, and surgical complications were documented.

### Statistical analysis

The conformity of continuous variables to a normal distribution was investigated by examining sample size, skewness-kurtosis values, and normality tests. None of the continuous variables were found to exhibit a normal distribution. Therefore, the Mann-Whitney U test was employed for independent group comparisons. Descriptive statistics, such as the median and interquartile range (IQR 25-75%) values were used. Categorical independent variables were displayed in cross-tabulations with frequencies and percentages. The comparison of variable distributions was conducted using both the Chi-Square Test and Fisher’s Exact Test methodologies. Box plot graphs were created for the CCD angle, and resultant shortening. Statistical analysis was performed using the SPSS 25.0 program (IBM, Armonk, New York, United States of America), and the significance level was determined as 0.05 in all analyses.

## Results

**Fig. 3:**
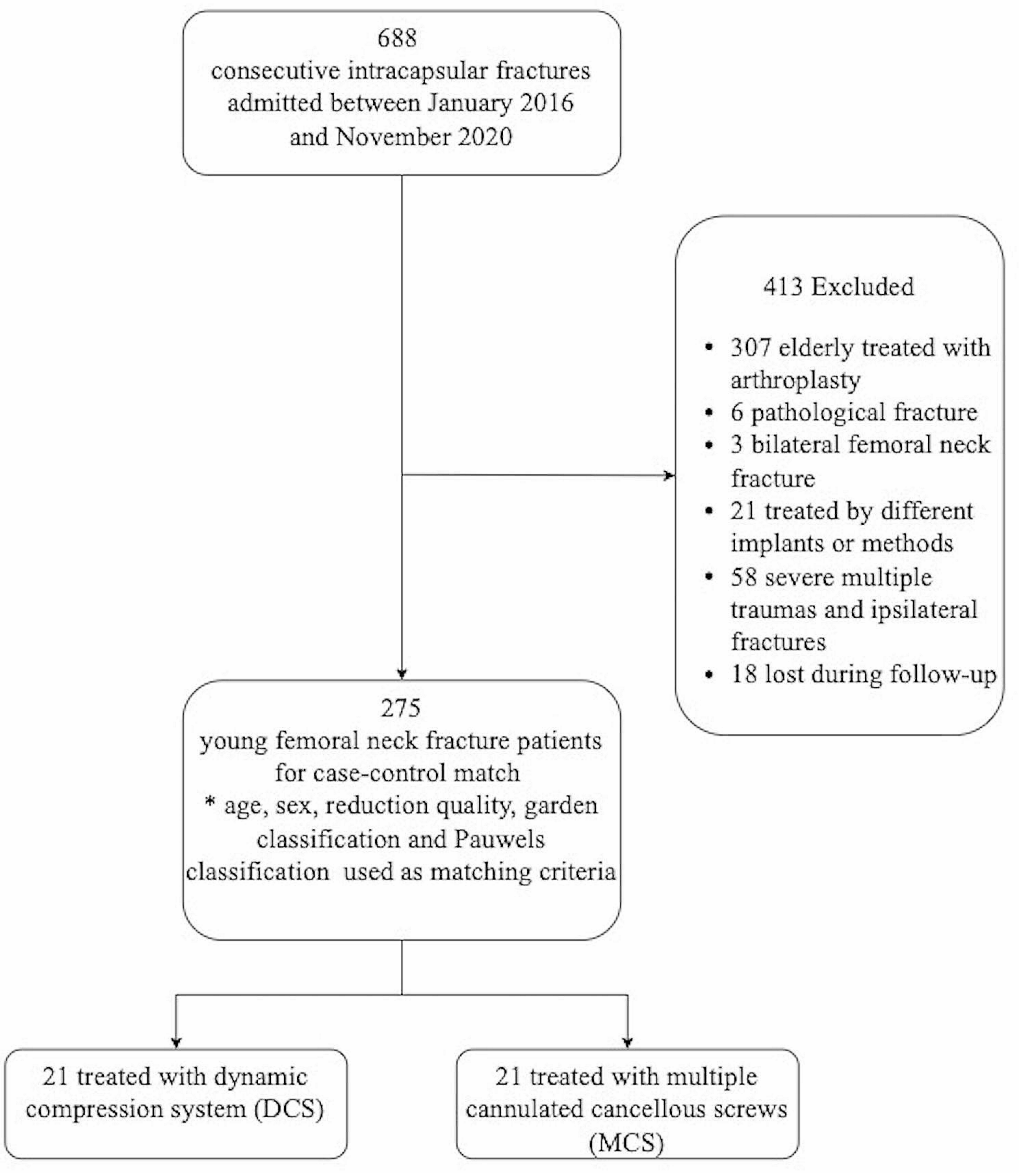
Flow diagram of the selection process for femoral neck fractures treated using the DCS or MCS implant

A total of 688 intracapsular hip fractures were identified from electronic records. According to the enrolment criteria discussed in the methods section, 413 patients were excluded: 307 underwent arthroplasty, 6 had pathological fractures, 3 had bilateral fractures, 21 were treated with other implants, 58 had multiple trauma injuries, and 18 were lost to follow-up. This left 275 patients included in the 1:1 case-control match (Fig. 3). 233 patients did not meet the matching criteria and were operated on using multiple cancellous screws. This study involved 42 confirmed cases of intracapsular hip fractures in patients of both sexes. Out of these, 21 cases were treated with DCS, and 21 with MCS. The median age in the DCS group was 41 years (range: 41–43), while the median age in the MCS group was 43 years (range: 41–46). The DCS group included 21 (57.1%) males and 9 (42.9%) females. The MCS group included 11 (52.4%) male patients and 10 (47.6%) female patients. The follow-up period for the DCS group ranged from 26 to 43 months, with a median of 30 months, and for the MCS group, it ranged from 27 to 51 months, with a median of 33 months. No significant differences were found between the two groups in terms of age, gender, or follow-up period (*p* > 0.05). Three fractures in each group were classified as non-displaced (Garden type I and II), while 17 fractures were classified as displaced (Garden type III and IV). Fourteen fractures in each group were categorized as Pauwels Grade II, and seven fractures as Pauwels Grade III. Excellent reduction was achieved in nine patients from each group, good reduction in eight patients, and fair reduction in four patients. The baseline characteristics and follow-up duration are detailed in Table 1.

**Table 1:** Patient characteristics

	DCS group	MCS group	*P* _value_	d_Cohen_
	Median (IQR)	Median (IQR)		
Age	41 (35 to 43)	43 (41 to 46)	0.210^*^	0.392
Follow-up period (month)	30 (26 to 43)	33 (27 to 51)	0.284^*^	0.335
Gender	n. (%)	n. (%)		
*Male*	12 (57.1%)	11 (52.4%)	1.000^**^	0.096
*Female*	9 (42.9%)	10 (47.6%)		
Side				
*Right*	7 (33.3%)	9 (42.9%)	0.751^**^	0.197
*Left*	14 (66.7%)	12 (57.1%)		
Garden classification				
*Type I*	2 (9.5%)	2 (9.5%)	na	na
*Type II*	2 (9.5%)	2 (9.5%)		
*Type III*	8 (38.1%)	8 (38.1%)		
*Type IV*	9 (42.9%)	9 (42.9%)		
Pauwels classification				
*Type II*	14 (66.7%)	14 (66.7%)	1.000^**^	0.000
*Type III*	7 (33.3%)	7 (33.3%)		
Reduction quality				
*Excellent*	9 (42.9%)	9 (42.9%)	na	na
*Good*	8 (38.1%)	8 (38.1%)		
*Fair*	4 (19%)	4 (19%)		

IQR; Interquartile range, *Mann-Whitney Test **Chi-Square - Fisher’s Exact Tests

**Fig. 4:**
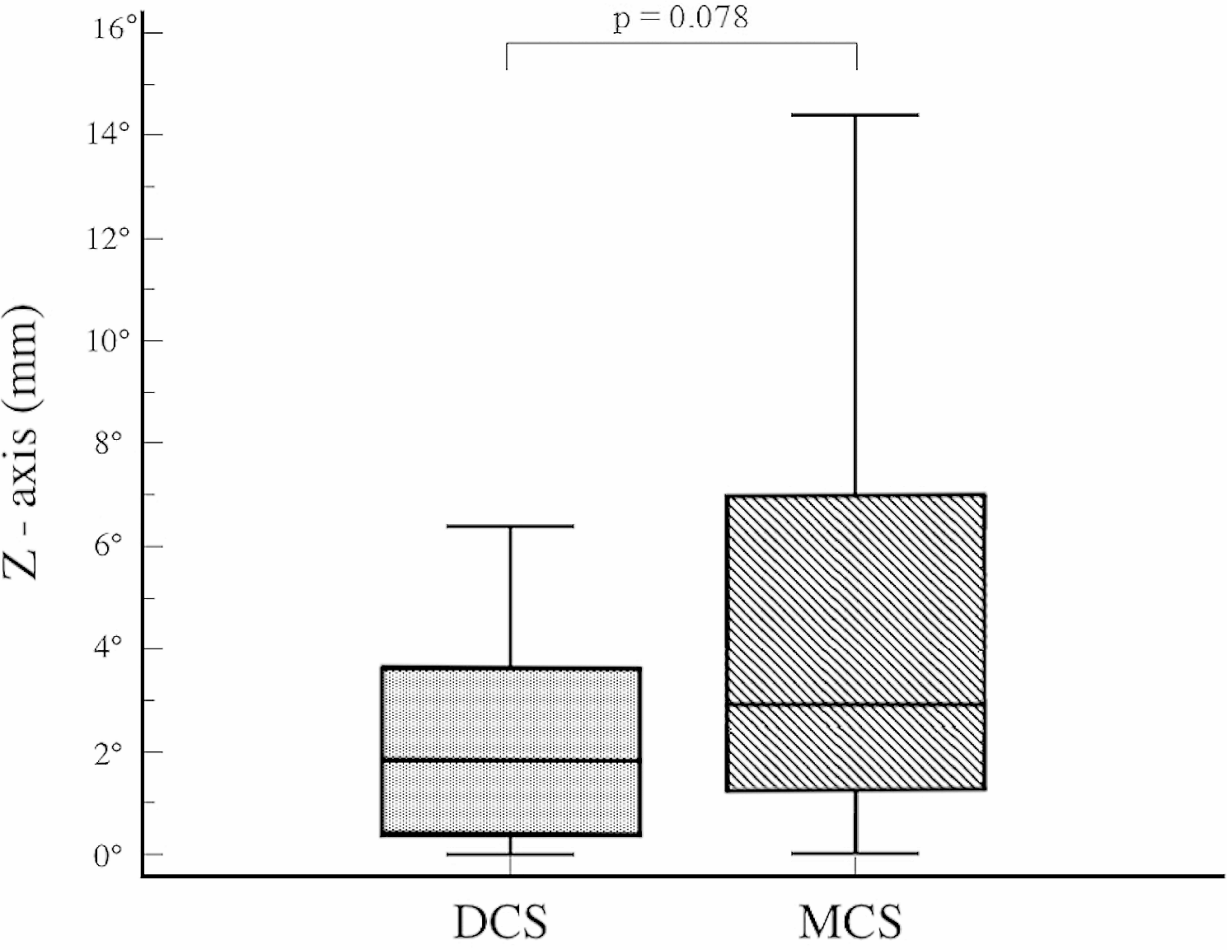
The amount of femoral neck shortening in the Z axis

**Fig. 5:**
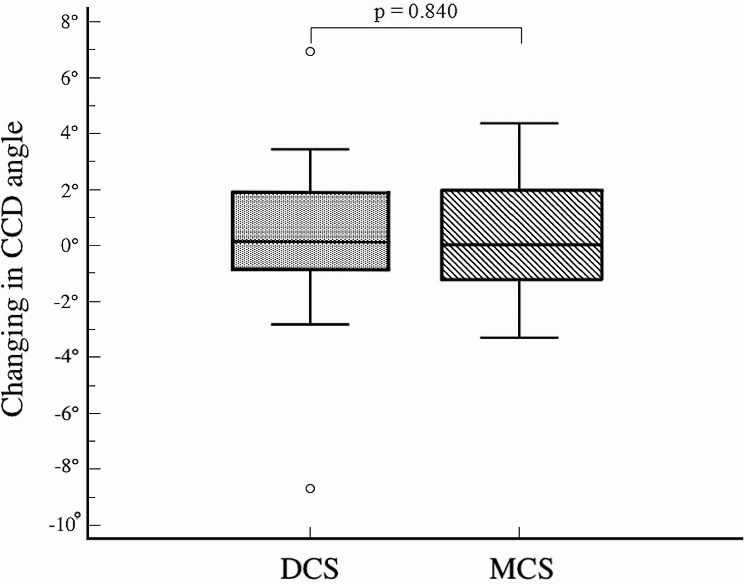
The amount of change in CCD angle at fracture union

A significant difference in femoral neck shortening was noted in the vertical plane (Y axis) (*p* = 0.004); however, no significant differences were observed in the horizontal plane (X axis) (*p* = 0.227), or in the resulting shortening (Z axis) (*p* = 0.078) (Fig. 4). No significant difference was observed in CCD angle measurements between both hips within the same group or between the two groups throughout the follow-up period (*p* > 0.05). The change in CCD angle during healing was found to be similar in both groups (*p* = 0.84) (Fig. 5). Clinically, no statistically significant differences were found in HHS, hospitalization duration, and operation time between the two groups (*P* = 0.89, *p* = 0.683, and *p* = 0.453, respectively) (Table 2).

**Table 2:** Clinical and radiological parameters

	DCS group	MCS group	*P*_Value*_	d_Cohen_
	Median (IQR)	Median (IQR)		
Operation time (minutes)	60 (55 to 70)	60 (55 to 70)	0.683	0.124
Hospitalization (days)	3 (2 to 4)	2 (2 to 3)	0.453	0.227
Harris Hip Score	91 (85 to 93)	91 (75 to 93)	0.890	0.043
Vertical shortening (Y axis, mm)	1.92 (0.50 to 3.09)	4.53 (1.7 to 8.49)	**0.004**	0.976
Horizontal shortening (X axis, mm)	0.57 (0.43 to 4.74)	1.00 (0.56 to 6.23)	0.227	0.379
Resultant shortening (Z axis, mm)	1.82 (0.40 to 3.53)	2.74 (1.59 to 6.71)	0.078	0.565
The amount of change in CCD angle during healing at fracture union	0.13° (-0.78° to 1.80°)	-0.18° (-1.11° to 1.85°)	0.840	0.062
CCD angle of the uninjured hip	134.4°(130.0° to 136.5°)	132.6°(131.0° to 135.7°)	0.615	0.156
CCD angle of the operated hip	132.1°(129.7° to 133.4°)	131.5°(128.4° to 134.4°)	0.870	0.050
*Related Samples P*_*Value*_^§^	*p* = 0.149 (d = 0.663)	*p* = 0.689 (d = 0.175)		

CCD; caput-collum-diaphysis, IQR; Interquartile range, *Mann-Whitney Test, ^§^: Wilcoxon Signed Ranks Test

Osteonecrosis of the femoral head was observed in 4 patients (19%) in the DCS group and in 5 patients (23.8%) in the MCS group. One patient (4.8%) in the DCS group experienced a periprosthetic fracture, while one patient (4.8%) in the MCS group experienced implant failure. All osteonecrotic hips were revised with total hip arthroplasty. The periprosthetic fracture was revised with a long proximal femoral nail, and the implant failure was revised with a DHS. No statistically significant differences in postoperative complication rates were observed (*p* > 0.05). No instances of pulmonary embolism, deep vein thrombosis, incision infection, surgical complications, or cerebrovascular and cardiovascular incidents were reported. Four fractures with posterior femoral neck cortical fragmentation were observed in each group (*p* > 0.05).

## Discussion

This paper outlines the outcomes observed in younger individuals who underwent treatment for femoral neck fractures using either DCS or MCS fixation with a subsequent follow-up period of 24 months. The vertical femoral neck shortening was significantly reduced by the DCS implant; however, no significant differences in the clinical and radiological outcomes were observed.

Internal osteosynthesis for femoral neck fractures involves the use of cancellous screws, dynamic fixed-angle, and static fixed-angle implants. Ensuring the healing of femoral neck fractures required achieving three essential principles of internal fixation: interfragmentary compression, resistance to displacement, and maintenance of rotational stability throughout the healing period. Dynamic devices enabled compression at the fracture line during weight-bearing, enhancing the healing of fractures. Static devices preserved the reduction quality obtained during surgery and proved rigid fixation during fracture healing [22]. The MCS implant provided a minimally invasive solution featuring anti-stress and anti-rotation capabilities. DHS had the function of dynamic compression and tension band properties; therefore, DHS maintained the neck-shaft angle and anatomical reduction throughout fracture healing [23]. The DHS implant demonstrated better stability compared to standard fixation methods used for unstable femoral neck fractures [24]. The FNS demonstrated superior stability compared to MCS, matching the stability of DHS. Additionally, its implantation procedure exhibited greater minimally invasive characteristics compared to DHS [25]. However, a recent clinical study reported no difference in complications or functional outcomes between DHS, FNS and MCS [9]. Randomised trials showed no superiority of single internal fixation over others, and the best choice remained controversial [6].

DCS has been reported as an effective fixation method for femoral neck fracture [10–12]. The concept of the DCS aims to amalgamate the benefits inherent in both MCS and DHS. The DCS device consists of three screws arranged parallelly in an inverted triangular configuration. This arrangement facilitates the dispersion of screws within the safe cross-sectional area of the femoral neck, particularly in regions of high bone density, ensuring optimal holding strength. The plate adds more stability to the cancellous screws, making the implant more resistant to shear and torsion [10].

Previous research findings indicated that the DHS group consistently exhibited higher levels of operative blood loss, incision length, and surgery duration when compared to the DCS group [12]. Shu et al. reported no significant difference in surgical blood loss, incision length and operation time between DCS and MCS [11]. Wang et al. reported a significant prolongation in both surgical duration and hospital admission period in the DCS group compared to the FNS group. However, no notable variance in intraoperative blood loss between the two implants was observed [26]. Xiao et al. reported the results of 36 elderly patients with femoral neck fractures treated with DCS, with average hospitalization duration and operation time being 15.33 ± 3.71 days and 50.25 ± 11.77 min, respectively [10]. The study included a young population without comorbidities, resulting in a shorter hospitalization duration compared to previous studies. Nevertheless, the operation time was relatively consistent with previous reports. In this study, both hospitalization duration and operation time were comparable; therefore, the DCS, similar to MCS, was deemed a minimally invasive and less time-consuming method.

Femoral neck shortening was widely accepted as a common occurrence after femoral neck fractures [9, 11]. A study by Zlowodzki et al. reported a femoral neck shortening rate of 27 to 31% following MCS fixation [17]. Femoral neck shortening has a significant negative impact on the patient’s physical functioning. Clinic studies showed a positive impact of DCS fixation on preserving femoral neck length compared to MCS and DHS [11, 12]. Xiao et al. observed femoral neck shortening of less than 5 mm in only three out of the 36 patients [10]. Wang et al. reported comparable results of femoral neck shortening between FNS and DCS [26]. A biomechanical study indicated that adding an interlocking plate to three screws reduced femoral head centre migrations by 1.6 mm, though this had limited clinical impact [27]. In this study, the results for femoral neck shortening were consistent with the previous studies, with a significant effect only in the vertical plane (Y axis). Basso et al. attributed shortening in the MCS group to varus rotation [27]. This evidence, along with the current CCD angle results, suggested that the DCS exhibited a limited effect on varus deformity.

HHS was recognized as the most reliable scoring system for evaluating clinical outcomes after femoral neck fractures. A direct correlation between HHS and post-operative complications was established [21]. Previous functional evaluations using the HHS showed superior clinical outcomes for DCS fixation compared to MCS and DHS [11, 12]. Xiao et al. reported excellent and good HHS rates in femoral neck fractures treated with DCS devices [10]. Wang et al. presented early results supporting FNS over DCS; however, no differences were observed after six months. A recent study found no significant difference between FNS, MCS and DHS [9]. This study’s results were consistent with previous reports, though no significant statistical difference in functional outcomes was noted.

In young individuals with femoral neck fractures, a relatively high incidence of complications has been observed. Osteonecrosis of the femoral head and non-union were commonly encountered complications that contributed significantly to unfavourable outcomes and often necessitating reoperations and salvage procedures [28]. Osteonecrosis of the femoral head has been associated with severe disturbances in vascularity around the femoral head, with reported incidences ranging from approximately 4.6–23% in cases of femoral neck fractures in young individuals [6, 28, 29]. Chang et al. reported that the incidence of osteonecrosis of the femoral head was approximately 3.8% following DCS osteosynthesis and 12.5% following DHS osteosynthesis [12]. No cases of osteonecrosis was reported by other authors in patients with Garden type II and III after fixation with a DCS implant [10, 11]. The initial displacement of the femoral neck fracture was identified as a significant risk factor for femoral head osteonecrosis [28]. A meta-analysis study reported the incidence of osteonecrosis in Garden grade III and grade IV were 17% and 32.8%, respectively [30]. In this study, osteonecrosis rates were reported as 23.8% for the DCS group and 19% for the MCS group. Displaced femoral fractures were observed more frequently than nondisplaced fractures in this study, resulting in a higher incidence of osteonecrosis compared to previous studies involving the DCS implant.

Displaced fracture and poor reduction have been recognized as predictive factors for non-union in femoral neck fracture patients [31]. The reported non-union rates for femoral neck fractures vary between 5% and 23% [4, 28, 29]. Chang et al. and Shu et al. reported a non-union rate of 3.8 to 7.1% in the DCS group, with no significant difference from the DHS and MCS groups [11, 12]. Xiao et al. and Wang et al. reported no instances of non-union in patients treated with the DCS implant [10, 26]. In this study, there were no fractures complicated with non-union. Previous studies used the locking feature of the DCS device; however, locking may diminish the dynamic compression feature of the device, potentially leading to non-union and screw migration through the femoral head, resulting in intra-articular penetration [32, 33].

This study reports, for the first time, a subtrochanteric fracture developed after treating a femoral neck fracture with a small-size DCS. Subtrochanteric fractures following MCS and FNS fixation of femoral neck fractures are recognized complications [34, 35]. Locking screws in the plate, which increases construct stiffness, or a screw start point distal to the lesser trochanter were associated with such complications [35, 36]. In this study, a subtrochanteric fracture developed due to the placement of an inappropriate size plate below the lesser trochanter. Proper selection of implant size is recommended to prevent such complications.

One limitation of this retrospective cohort study was its small sample size. Comparison of healing times between the two groups was not conducted due to constraints on the number of available follow-up time points. Surgeon discretion in selecting the fixation device may have introduced selection bias; however, a 1:1 pairing method was employed to mitigate this bias. The HHS was conducted at the last follow-up, limiting the evaluation of early functional outcomes. Additionally, screws in the DCS group were intentionally left unlocked, potentially affecting device stability, although femoral neck shortening was less than 5 mm in all planes. Consequently, the outcomes of this study necessitate additional confirmation through a prospective randomized controlled trial involving multiple devices, a substantial sample size, and participation from multiple centres.

## Conclusion

This study offered insights into the comparable clinical and radiological outcomes of DCS and MCS fixation methods for femoral neck fractures in young adults. The DCS implant emerged as a viable alternative to MCS for treating femoral neck fractures. The addition of a plate to MCS was noted to enhance stability in the vertical axis without substantially prolonging surgical duration. However, functional outcomes and complication rates were similar between the two implants. The occurrence of a subtrochanteric fracture in the DCS group highlighted the critical importance of selecting appropriate implant sizes.

### Electronic supplementary material

Below is the link to the electronic supplementary material.

Supplementary Material 1

Supplementary Material 2

## Data Availability

No datasets were generated or analysed during the current study.
