# Supplementary material for: Comparison of dynamic compression system versus multiple cancellous screws in the treatment of femoral neck fractures in young adults
Source: J Orthop Surg Res. 2024 Jul 22;19:422. doi: 10.1186/s13018-024-04913-7 (PMC11265074; doi:10.1186/s13018-024-04913-7)
Supplement: Supplementary file 2 — Supplementary Material 2 [file 13018_2024_4913_MOESM2_ESM.pdf]

STROBE Statement—Checklist of items that should be included in reports of ***case-control studies***

	Item No	Recommendation	
**Title and abstract**	1	(*a*) Indicate the study's design with a commonly used term in the title or the abstract	Page 1 Abstract Materials and Methods section.
		(*b*) Provide in the abstract an informative and balanced summary of what was done and what was found	Informative summary had been provided in page 1
**Introduction**			
Background/rationale	2	Explain the scientific background and rationale for the investigation being reported	Provided in page 2 and page 3
Objectives	3	State specific objectives, including any prespecified hypotheses	Provided in page 3
**Methods**			
Study design	4	Present key elements of study design early in the paper	Provided in page 3 and 4
Setting	5	Describe the setting, locations, and relevant dates, including periods of recruitment, exposure, follow-up, and data collection	Provided in page 5 and 6
Participants	6	(*a*) Give the eligibility criteria, and the sources and methods of case ascertainment and control selection. Give the rationale for the choice of cases and controls Provided in page 3 and 4	
		(*b*) For matched studies, give matching criteria and the number of controls per case Provided in page 4	
Variables	7	Clearly define all outcomes, exposures, predictors, potential confounders, and effect modifiers. Give diagnostic criteria, if applicable Provided in page 6 and 7	
Data sources/ measurement	8*	For each variable of interest, give sources of data and details of methods of assessment (measurement). Describe comparability of assessment methods if there is more than one group Provided in page 4, 6 and 7	
Bias	9	Describe any efforts to address potential sources of bias Provided in page 4	
Study size	10	Explain how the study size was arrived at Provided in page 4	
Quantitative variables	11	Explain how quantitative variables were handled in the analyses. If applicable, describe which groupings were chosen and why Provided in page 7	
Statistical methods	12	(*a*) Describe all statistical methods, including those used to control for confounding	
		(b) Describe any methods used to examine subgroups and interactions	
		(c) Explain how missing data were addressed	
		(d) If applicable, explain how matching of cases and controls was addressed	
		(*e*) Describe any sensitivity analyses Provided in page 7	
**Results**			

Participants	13*	(a) Report numbers of individuals at each stage of study—eg numbers potentially eligible, examined for eligibility, confirmed eligible, included in the study, completing follow-up, and analysed Provided in page 8
		(b) Give reasons for non-participation at
		(c) Consider use of a flow
		Provided in page 7 and 8 Provided in page 7 and
Descriptive data	14*	(a) Give characteristics of study participants (eg demographic, clinical, social) and information on exposures and potential confounders Provided in page 7 and 8
		(b) Indicate number of participants with missing data for each variable of interest No missing data
Outcome data	15*	Report numbers in each exposure category, or summary measures of exposure Provided in page 7 and 8
Main results	16	(*a*) Give unadjusted estimates and, if applicable, confounder-adjusted estimates and their precision (eg, 95% confidence interval). Make clear which confounders were adjusted for and why they were included NA
		(*b*) Report category boundaries when continuous variables were categorized Provided in page 7- 8 and table 1
		(*c*) If relevant, consider translating estimates of relative risk into absolute risk for a meaningful time period NA

Other analyses	17	Report other analyses done—eg analyses of subgroups and interactions, and sensitivity analyses NA
**Discussion**		
Key results	18	Summarise key results with reference to study objectives Provided in page 9
Limitations	19	Discuss limitations of the study, taking into account sources of potential bias or imprecision. Discuss both direction and magnitude of any potential bias Provided in page 13
Interpretation	20	Give a cautious overall interpretation of results considering objectives, limitations, multiplicity of analyses, results from similar studies, and other relevant evidence Provided in page 10-12
Generalisability	21	Discuss the generalisability (external validity) of the study results Provided in page 10-13
**Other information**		
Funding	22	Give the source of funding and the role of the funders for the present study and, if applicable, for the original study on which the present article is based Provided in page 14

*Give information separately for cases and controls.

Note: An Explanation and Elaboration article discusses each checklist item and gives methodological background and published examples of transparent reporting. The STROBE checklist is best used in conjunction with this article (freely available on the Web sites of PLoS Medicine at http://www.plosmedicine.org/, Annals of Internal Medicine at http://www.annals.org/, and Epidemiology at http://www.epidem.com/). Information on the STROBE Initiative is available at http://www.strobe-statement.org.
